# The C-peptide Signaling

**DOI:** 10.1080/15438600490424497

**Published:** 2004

**Authors:** George Grunberger, Anders A. F. Sima

**Affiliations:** 1 Grunberger Diabetes Institute Bloomfield Hills Michigan USA; 2 Departments of Pathology and Neurology and Morris Hood Jr.Comprehensive Diabetes Center Wayne State University Detroit Michigan USA; 3 Diabetes Institute 43494 Woodward Avenue Suite 208 Bloomfield Hills MI 48302 USA

## Abstract

For years an assumption was made that C-peptide, a byproduct of insulin biosynthesis, possessed no appreciable physiologic role. As other contributions in this volume amply testify, the time has come to re-evaluate that notion. C-peptide either directly through interaction with its specific cell-surface receptor or indirectly through an interaction with a related membrane entity, exerts a unique effect on several intracellular processes.We review here results of studies attempting to elucidate such molecular effects of C-peptide in different cell systems and tissues. Lacking a purified C-peptide receptor, we also demonstrate C-peptide effects on distinct elements of the insulin signal transduction pathways.


*Experimental Diab. Res.*, 5:25–36, 2004
Copyright © Taylor and Francis Inc.
ISSN: 1543-8600 print / 1543-8619 online
DOI: 10.1080/15438600490424497

Taylor & Francis
Taylor & Francis Group

### The C-peptide Signaling

**George Grunberger[1] and Anders A. F. Sima[2]**

[1]*Grunberger Diabetes Institute, Bloomfield Hills, Michigan, USA*
[2]*Departments of Pathology and Neurology and Morris Hood Jr. Comprehensive Diabetes Center, Wayne State University, Detroit, Michigan, USA*

**For years an assumption was made that C-peptide, a byproduct of insulin biosynthesis, possessed no appreciable physiologic role. As other contributions in this volume amply testify, the time has come to re-evaluate that notion. C-peptide either directly through interaction with its specific cell-surface receptor or indirectly through an interaction with a related membrane entity, exerts a unique effect on several intracellular processes. We review here results of studies attempting to elucidate such molecular effects of C-peptide in different cell systems and tissues. Lacking a purified C-peptide receptor, we also demonstrate C-peptide effects on distinct elements of the insulin signal transduction pathways.**

**Keywords** 3T3 Fibroblasts; C-Peptide Receptor; C-Peptide Signal Transduction; Insulin Signal Transduction; L6 Myoblasts; LEII Mouse Lung Cells; Neuronal Apoptosis; Skeletal Muscle Cells

### INTRODUCTION AND BACKGROUND

C-peptide was first described in 1967 as a byproduct of insulin biosynthesis (Steiner, 1967). It had been accepted for years that it was just an inert molecule with no intrinsic physiological role. That view was fueled by investigators' inability to demonstrate any apparent biological activity in assays used (such as lowering of blood glucose concentration), by a perceived lack of a specific cell-surface peptide receptor, and by the seemingly satisfactory explanation of C-peptide's role as a substance allowing proper folding of the proinsulin molecule. That view has undergone transformation over the past decade as data have accumulated showing diverse biological effects after administration of C-peptide. These results are described in detail elsewhere in this volume. To summarize, in several animal models of diabetes and in patients with autoimmune insulin-deficient (type 1) diabetes mellitus, C-peptide replacement seems to enhance skin and skeletal muscle blood flow, to improve renal function, to ameliorate sensory nerve dysfunction, to accelerate sural and peroneal nerve conduction velocity, to improve autonomic nerve function, to augment glucose utilization, and to ameliorate impaired deformability of erythrocytes.

Although the decade of 1990s showed conclusively that C-peptide has biological effects, it remains for the 2000s to settle the question of specific molecular mechanism(s) responsible for these observations.

It has been accepted for several decades that for any peptide hormone to exert specific cellular effects, a specific receptor should be identifiable at the cell surface. Typically, the hormone binds in a saturable, reversible manner and activates specific intracellular events. Identifying such a specific receptor for proinsulin C-peptide has proven elusive. As a result, many of the previously shown effects of C-peptide were instead explained by nonchiral membrane interactions (Ido et al., 1997). It was not until 1999 when the first credible report identified specific C-peptide binding to cell membranes in cultured renal tubular cells, and saphenous vein endothelial cells (Rigler et al., 1999). The technique used in that report, fluorescence correlation spectroscopy, is apparently sensitive enough to demonstrate the presence of specific ligand-receptor interactions where the traditional radioligand methods had previously failed.

Received 11 September 2003; accepted 28 November 2003.
These studies were supported by the Henry L. Brasza Endowment (GG), the Juvenile Diabetes Research Foundation (AAFS), the Morris Hood Jr. Comprehensive Diabetes Center (GG, AAFS), and the Thomas Foundation (AAFS).
Address correspondence to Dr. George Grunberger, Grunberger Diabetes Institute, 43494 Woodward Avenue, Suite 208, Bloomfield Hills, MI 48302, USA.

The question that needs to be settled is whether the unequivocally observed biological effects of C-peptide are mediated by the peptide's direct and specific interaction with a cognate receptor or indirectly through and interaction with a receptor for different ligand(s).

What have been the intracellular effects of C-peptide that call for investigations of their molecular underpinnings? C-peptide, for example, stimulates glucose transport in human skeletal muscle (Zierath et al., 1996) in a dose-dependent fashion. In L-6 myoblasts, C-peptide increases glycogen synthesis and amino acid uptake (Grunberger et al., 2001). Early on, C-peptide was found to enhance $Na^+$,$K^+$-ATPase activity in rat sciatic nerve, granulation tissue, red cells, pancreatic islets, renal cells, and other tissues. Further, C-peptide activates endothelial nitric oxide synthase, resulting in release of nitric oxide from bovine aortic endothelial cells (Wahren et al., 2000). These 2 mechanisms have been accepted as the underlying principles of C-peptide's biological effects. However, moving from the physiological observations to the intracellular enzymology still begs the questions as to what is the proximal specific molecular mechanism which C-peptide triggers upon encountering the cell membrane.

### NATURE OF THE C-PEPTIDE RECEPTOR

Rigler et al. (1999), in their above-referenced paper, which identified C-peptide binding to human cell membranes, reported an inhibitory effect of pertussis toxin on the specific interaction of C-peptide with the cell surface. This finding led to their conclusion that the C-peptide–stimulated $Na^+$,$K^+$-ATPase activity involved G proteins. They postulated that a G protein interacting with a ligand-activated receptor might constitute an allosteric system. Pertussis toxin might then interfere with interaction between the G protein and the loop regions of the membrane-spanning receptor. These results were consistent with previous reports showing that pertussis toxin and protein phosphatase 2B (calcineurin) inhibitor abolished $Na^+$,$K^+$-ATPase activity in rat renal tubular segments (Ohtomo et al., 1996). A possible scenario of C-peptide signaling would then include activation of a specific pertussis toxin-sensitive G protein–coupled receptor and activation of a calcium-dependent intracellular signaling.

### C-PEPTIDE SIGNAL TRANSDUCTION IN HUMAN SKELETAL MUSCLE

The initial attempt to answer the question was published by Zierath et al. (1996). They studied the molecular basis of C-peptide–stimulated glucose transport in human skeletal muscle. Isolated muscle tissue was obtained by open biopsy from the vastus lateralis portion of the quadriceps muscle from healthy and type 1 diabetic subjects. They reported that both insulin (6 nmol/L) and C-peptide (2.5 nmol/L) increased 3-$O$-methylglucose transport about 2-fold. Interestingly, coincubation of the muscle strips with both insulin and C-peptide at the above physiological concentrations resulted in the same degree of stimulation as with either hormone alone. In view of the nonadditivity of the peptide affects, the authors concluded that C-peptide likely activates glucose transport in human skeletal muscle via an insulin-mediated system. Thus, they set up to investigate whether C-peptide interacted with the insulin receptor and the proximal parts of the insulin-signaling pathway. They could not demonstrate specific binding of radiolabeled C-peptide to crude membranes prepared from the muscle, nor tyrosine kinase activity of partially purified insulin receptors from that tissue. Differences in C-peptide– and insulin-mediated signaling were inferred from the results of studies using isoproterenol, a $\beta$-adrenergic agonist. Isoproterenol inhibited insulin-stimulated but not the C-peptide–stimulated glucose transport. On the other hand, the c-AMP analog, $Bt_2cAMP$, abolished both insulin- and C-peptide–stimulated effects. The authors' results thus left the molecular mechanism of the C-peptide's insulinomimetic effect unanswered. They concluded that a possible explanation for the nonadditivity of C-peptide and insulin effects, in the absence of C-peptide–activated insulin receptor binding and tyrosine kinase activity, might be signaling pathways shared by the 2 hormones distal to the tyrosine kinase activation.

### C-PEPTIDE SIGNALING IN SWISS 3T3 FIBROBLASTS AND 3T3-F442A CELLS

The hypothesis was pursued by Kitamura et al. (2001). They showed that human and rat (1 and 2) C-peptide stimulated phosphorylation of mitogen-activated protein kinase (MAPK) in Swiss 3T3 fibroblasts and 3T3-F442A cells. Interestingly, they failed to see the same effect in other cells (3T3-L1 fibroblasts, HepG2 hepatoma, C6 glioma, $L_6E_9$muscle, NG108.15 neuroblastoma, and $GH_4C_1$ somatomamotrophic cells), emphasizing the importance of cell specificity and the danger of simple extrapolation of data from one system to another. In the responsive cells, C-peptide increased MAPK phosphorylation at concentrations between 1 pM and 1 nM (half maximal dose was 0.25 nM for p44 MAPK and 0.28 nM for p42 MAPK). Higher concentrations of C-peptide were not effective and there was no increase in total MAPK concentrations induced by any dose of C-peptide. Insulin was used at 100 times higher concentration (100 nM) and had 2-fold higher maximum effect on MAPK phosphorylation than C-peptide. The investigators, unfortunately, did not assess simultaneous effects of insulin and C-peptide to answer the issue of additivity of the hormones' actions. In more detailed pursuit of mechanism of C-peptide effect, C-peptide was found to phosphorylate mitogen-activated protein kinase kinase (MAPKK) (MEK), an enzyme that phosphorylates and activates MAPK. PD98059, a MEK inhibitor, abolished that stimulatory action of C-peptide. Pertussis toxin preincubation of cells led to inhibition of C-peptide–stimulated MAPK phosphorylation. The authors concluded that C-peptide activates MAPK through a $G_i/G_0$-linked receptor. In investigating additional potential signaling pathways activated by C-peptide, Kitamura et al. (2001) assessed a possible effect of wortmannin, a phosphoinositol 3-kinase (PI3K) inhibitor on C-peptide–stimulated MAPK phosphorylation. Wortmannin inhibited the C-peptide effect. Similarly, GF 109203X, a protein kinase C inhibitor, abolished the C-peptide–induced MAPK phosphorylation. Pretreatment of cells with phorbolmyristate acetate (PMA) down-regulated protein kinase C activity. It also inhibited the ability of C-peptide to stimulate MAPK phosphorylation in such cells. From these experiments, C-peptide would thus seem to mediate its cellular effects via PI3K-dependent and protein kinase C–dependent pathways.

### C-PEPTIDE SIGNALING IN LEII MOUSE LUNG ENDOTHELIAL CELLS

The same authors (Kitamura et al., 2002) also examined effects of C-peptide in LEII mouse lung capillary endothelial cells. They demonstrated that human C-peptide (at 10 nM) induced an increase of ERK 1/2 phosphorylation (just as insulin did, at 100 nM). C-peptide also led to site-specific phosphorylation of p38 MAPK (Thr-183/Tyr-185), whereas insulin did not. C-peptide (1 nM)-induced phosphorylation of ERK 1/2 and p38 MAPK led to enhanced kinase activities of these enzymes (approximately 3-fold). This effect of human C-peptide was replicated using rat C-peptides 1 and 2; neither retrosequenced nor all D-amino acid human C-peptide induced phosphorylation of these kinases. To pursue the sequence of events induced by incubation of LEII cells with C-peptide, the authors also showed that phosphorylation of the cAMP response element-binding protein (CREB), activating transcription factor (ATF)1, and ATF2 (but not that of c-Jun, a substrate of c-Jun N-terminal kinase [JNK]) were increased. The C-peptide–induced phosphorylation was confirmed to promote the binding of CREB/ATF family proteins to DNA. In order to determine the involvement of ERK1/2 and p38 MAPK in the observed C-peptide effects on phosphorylation of CREB/ATF, the authors used several specific inhibitors. SB203580, a p38 MAPK inhibitor, abolished the C-peptide–induced phosphorylation of CREB/ATF and activation of p38 MAPK. PD98059, a MEK inhibitor, in contrast, had no such effect. Because there are intermediate steps between activation of p38 MAPK and phosphorylation of CREB/ATF, Kitamura et al. (2002) assessed activation of mitogen- and stress-activated protein kinase (MSK1), MAPKAP-K2, and ribosomal S6 kinase (RSK), all CREB kinases. C-peptide increased phosphorylation of MAPKAP-K2 and RSK. In turn, SB203580 (but not PD98059) inhibited this effect. Thus, it appears that C-peptide enhanced p38 MAPK followed by MAPKAP-K2 to increase interactions between CREB/ATF1 and DNA. The effects of C-peptide in the LEII cells were not identical to those of insulin. Insulin induced phosphorylation of ERK 1/2 but not that of p38 MAPK.

### C-PEPTIDE SIGNALING IN RAT MEDULLARY ASCENDING LIMB OF HENLE'S LOOP

Tsimaratos et al. (2003) studied the molecular mechanisms underlying C-peptide–stimulated $Na^+,K^+$-ATPase activity in isolated rat medullary thick ascending limb of Henle's loop. C-peptide–mediated increases in the enzyme activity was observed at physiological concentrations between 1 and 100 nM, with maximal effect at 100 nM at 37°C within 5 minutes of incubation of the medullary segments with C-peptide. The ATPase stimulation by C-peptide reached a plateau after 10 minutes. The scrambled sequence of C-peptide did not alter $Na^+,K^+$-ATPase activity. Apparently, the C-peptide effect was caused by increased turnover of the enzyme in this cell system. The stimulation of the ATPase activity was associated with enhanced phosphorylation of its $\alpha$-subunit. C-peptide, at 100 nM, stimulated the appearance of the $\alpha$-isoform of protein kinase C (PKC) in the membrane fraction. The amounts of other PKC fractions assessed in these studies were not affected by C-peptide preincubation of the segments. GF109203X, a specific PKC inhibitor, abolished the stimulatory effect of 100 nM C-peptide. The authors, therefore, concluded that the observed stimulation of $Na^+,K^+$-ATPase catalytic $\alpha$-subunit activity is PKC-$\alpha$ dependent.

### MOLECULAR BASIS FOR THE INSULINOMIMETIC EFFECTS OF C-PEPTIDE IN RAT SKELETAL MUSCLE CELLS

Grunberger et al. (2001) showed that synthetic rat C-peptide stimulates several insulin-like cellular effects in a rat skeletal muscle cell system. To elucidate the molecular mechanisms(s) underpinning such insulinomimetic actions as glycogen synthesis and amino acid uptake, they investigated the effects of C-peptide on several elements of the insulin signaling pathway.

Physiologic effects of insulin are initiated by insulin binding to the extracellular domain of the insulin receptor (IR). Binding results in activation of the IR tyrosine kinase (TKA), followed by phosphorylation of intracellular substrates, propagating receptor signals throughout the cell. These include the Ras/MAPK cascade and the PI3K/Akt (protein kinase B, PKB) system, which are thought to play key roles in the mitogenic and metabolic arms of insulin signaling, respectively (Holman and Kasuga, 1997; Virkamäki et al., 1999). C-peptide enhances insulin-stimulated IR autophosphorylation and TKA in vitro (Sima et al., 1998). Based on those observations, it was hypothesized that C-peptide could affect other downstream elements of insulin signal transduction pathways.

Preincubation with rat-II C-peptide led to a 2.5-fold increase in glycogen synthesis in L6 rat myoblasts. The dose-dependent effect of C-peptide showed a bell-shaped response (Figure 1*A*). Maximum effect was observed between 1 and 3 nM of C-peptide. Interestingly, it required 10 nM of insulin to achieve the 2-fold stimulation seen with 0.3 nM of C-peptide. A scrambled C-peptide sequence (random arrangement of the 31 amino acids of C-peptide), serving as a control, did not increase glycogen synthesis above basal level. Human C-peptide increased glycogen synthesis to the same degree as the homologous (rat) C-peptide. Combinations of submaximally effective insulin (10 nM) and submaximal concentrations of C-peptide (0.1 or 0.3 nM) increased glycogen synthesis over that achieved with either ligand alone (Figure 1*B*). Combinations of maximally effective concentrations of insulin and C-peptide, however, were not additive.

C-peptide (1 to 10 nM) stimulated amino acid uptake more than 2-fold in L6 myoblasts, an effect similar to that of 10 nM of insulin (Figure 2*A*). Statistically significant stimulation was documented between 0.3 nM and 10 nM of C-peptide.

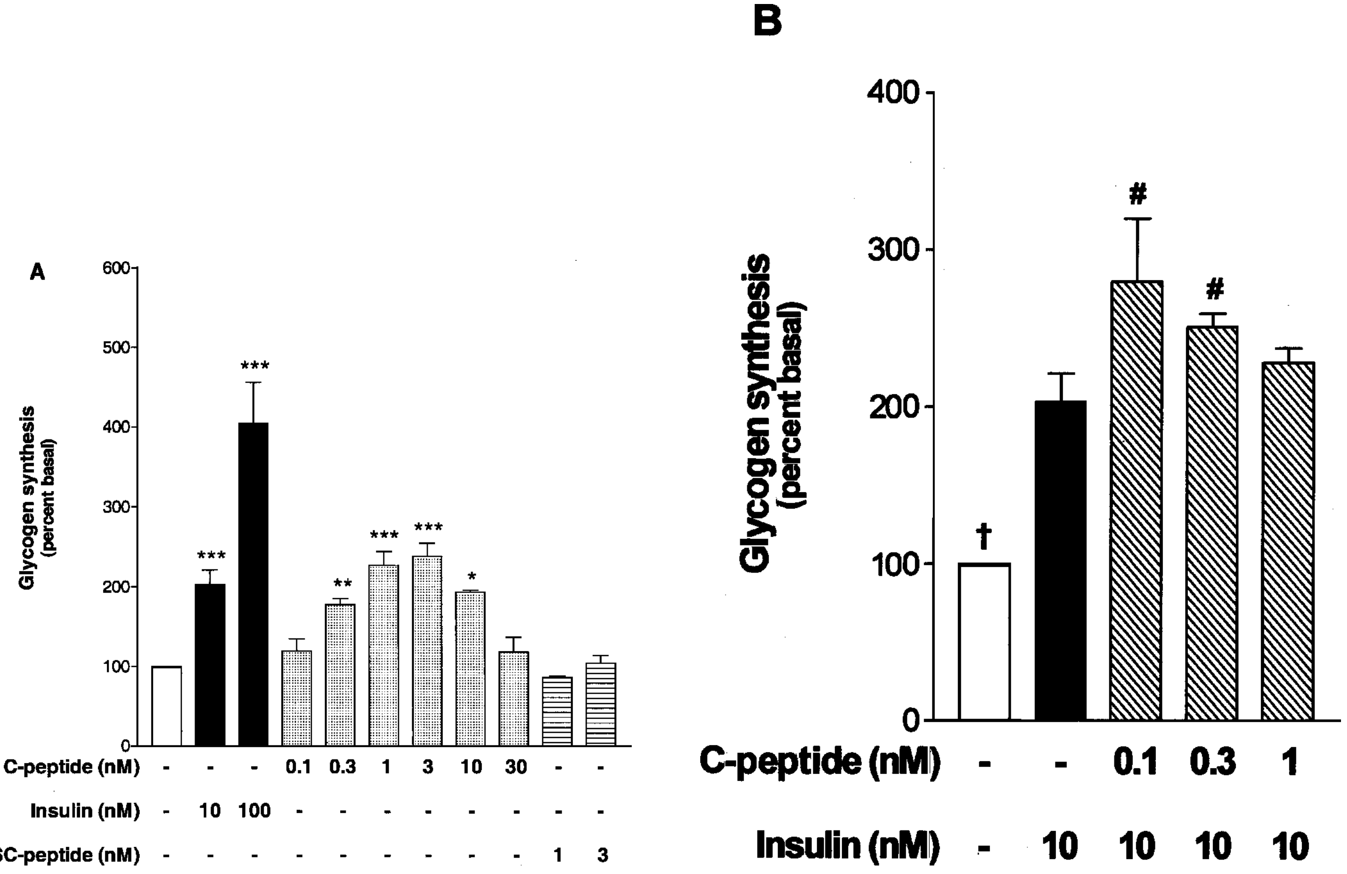

FIGURE 1

Stimulation of glycogen synthesis by C-peptide in L6 myoblasts. (*A*) L6 cells were serum-starved for 16 hours and then incubated with insulin, C-peptide, or scrambled C-peptide sequence for 1 hour. (*B*) L6 cells were serum-starved for 16 hours and then incubated with a combination of insulin and C-peptide for 1 hour. Results seen with 10 nmol/L insulin are shown for comparison. Glycogen synthesis was assessed by measuring D-[$^{14}$C]glucose incorporation into glycogen. Results are plotted as mean ± SEM of 10 separate experiments (each done in quadruplicate) and normalized with respect to control (=100%). Reproduced from Grunberger et al., 2001, by permission of *Diabetologia*. $^{***}P < .001$, $^{**}P < .01$, $^{*}P < .05$ versus control (*A*); and $^{\dagger}P < .05$ versus every sample, $^{\#}P < .05$ versus insulin (10 nmol/L) (*B*).

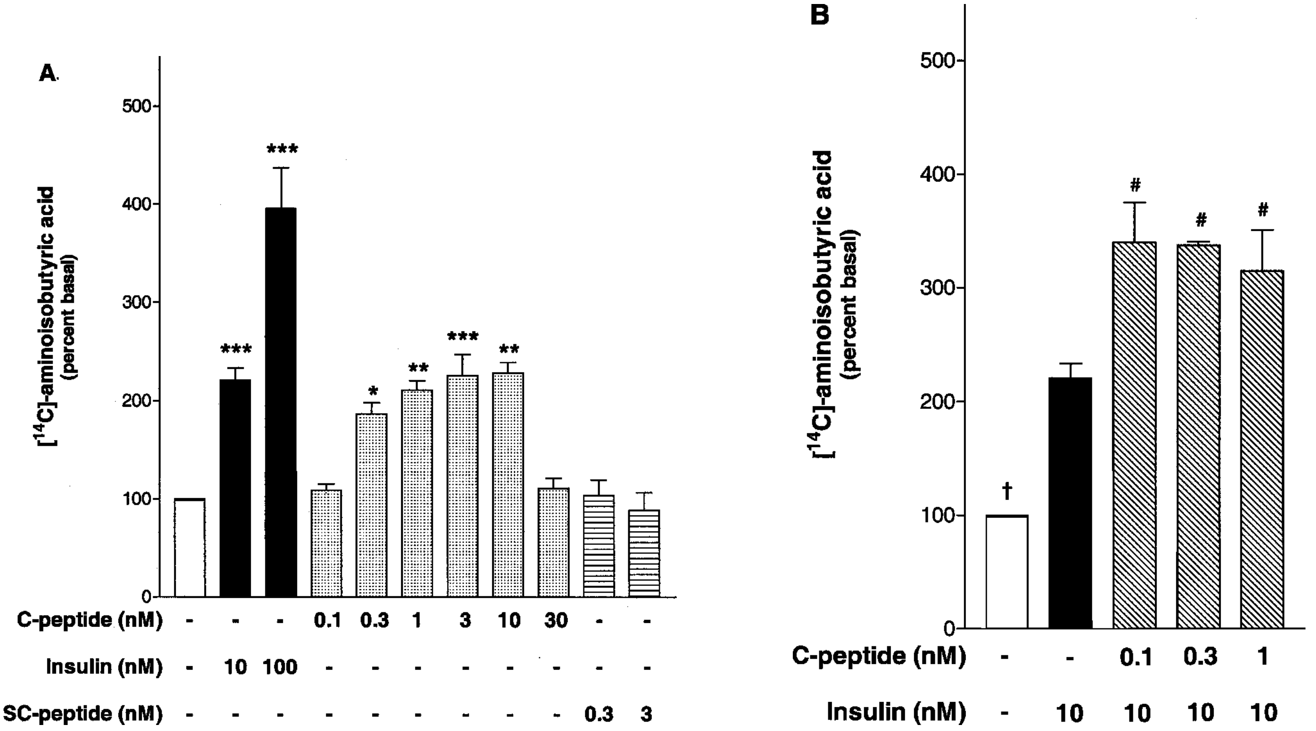

**FIGURE 2**

Stimulation of $[^{14}C]$aminoisobutyric acid uptake in L6 myoblasts. (*A*) L6 cells were serum-starved for 16 hours and then incubated with insulin, C-peptide, or a scrambled C-peptide sequence (SC-peptide) for 1 hour. (*B*) L6 cells were serum-starved and then incubated with a combination of insulin and C-peptide for 1 hour. Results obtained with 10 nmol/L insulin are shown for comparison. Amino acid uptake was measured by $[^{14}C]$methylaminoisobutyric acid incorporation. Results are presented as mean ± SEM of 3 separate experiments. Reproduced from Grunberger et al., 2001, by permission of *Diabetologia*. $^{***}P < .001$, $^{**}P < .01$, $^{*}P < .05$ versus control (*A*), $^{\dagger}P < .05$ versus every sample, $^{\#}P < .05$ versus insulin, 10 nmol/L.

Incubation of the cells with scrambled C-peptide did not increase the aminoisobutyric acid uptake over baseline. Addition of C-peptide (0.1 to 1 nM) to the submaximally effective insulin concentration (10 nM) significantly increased the amino acid uptake achieved with insulin alone (Figure 2*B*).

Based on the insulinomimetic effect of C-peptide on glycogen synthesis, the molecular basis for this observation was pursued. Insulin exerts its stimulatory effect on glycogen synthase by both activating protein phosphatase 1 and by inhibiting glycogen synthase kinase (GSK3), which is inactivated by phosphorylation (Halse et al., 1999). Preincubation of the cells with physiological concentrations of C-peptide (0.3 to 3 nM) led to increased GSK3 phosphorylation (7.5- and 5-fold, respectively) (Figure 3). The combinations of 10 nM insulin with either 0.1 or 0.3 nM C-peptide showed statistically significant increases in GSK3 phosphorylation over the effect of either hormone alone. C-peptide thus stimulates glycogen synthesis, at least partly, by inhibition of GSK3. Clearly, modulation of GSK3 is not the only mechanism by which glycogen synthesis is stimulated.

Insulin activates phosphorylation of Akt in L6 cells. Contrary to expectations raised by the insulinomimetic effects of C-peptide on glycogen synthesis, amino acid uptake, and GSK3 phosphorylation, C-peptide (at concentrations of up to 30 nM) did not stimulate phosphorylation of Akt whether it was assessed by immunoprecipitating or by immunoblotting with an anti–phospho-Akt antibody (Thr-308). Further, in a separate study, intact L6 myotubes were labeled with $^{32}P$-orthophosphoric acid for 6 hours, and stimulated with insulin, with C-peptide (0.3, 3 nM), with scrambled C-peptide (0.3 nM), or a combination of insulin and C-peptide. Cell lysates were immunoprecipitated with anti-Akt 1 antibody. Insulin (10 nM) stimulated phosphorylation of Akt 3.1-fold, but C-peptide did not. These data indicate that the observed insulinomimetic biological effects of C-peptide (Figures 1 and 2) likely involve divergent signaling pathways proximal to Akt activation.

Incubation of L6 myoblasts with C-peptide, with concentrations as low as 0.3 nM, also led to stimulation of Rsk phosphorylation (2.6-fold). Activation of Rsk was also ascertained by assessing its enzyme activity, using the p70S6 Rsk substrate (RRRLSSLRA). C-peptide, at 0.3 and 3 nM, activated Rsk as did insulin (10 and 100 nM) in L6 myoblasts. Combination of submaximally effective insulin concentration (10 nM)

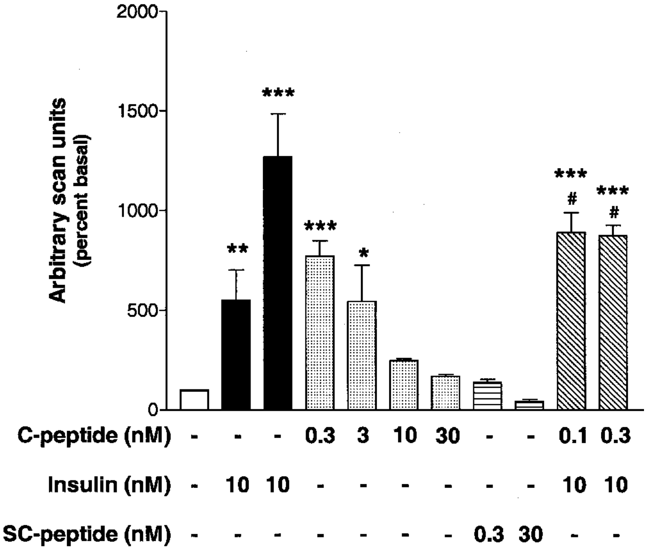

**FIGURE 3**

Stimulation of GSK-3 phosphorylation by C-peptide. Serum-starved confluent L6 myoblasts were incubated with insulin, C-peptide, or scrambled C-peptide sequence for 10 minutes. Cell lysates were separated by 7.5% SDS-PAGE, transferred to nitrocellulose membrane, and immunoblotted with anti–phospho-GSK3 antibody. Data from 4 separate experiments are plotted as mean ± SEM for each condition normalized for control (=100%). Reproduced from Grunberger et al., 2001, by permission of *Diabetologia*. $^{***}P < .001$, $^{**}P < .01$, $^{*}P < .05$ versus control; $^{\#}P < .05$ versus insulin (10 nmol/L).

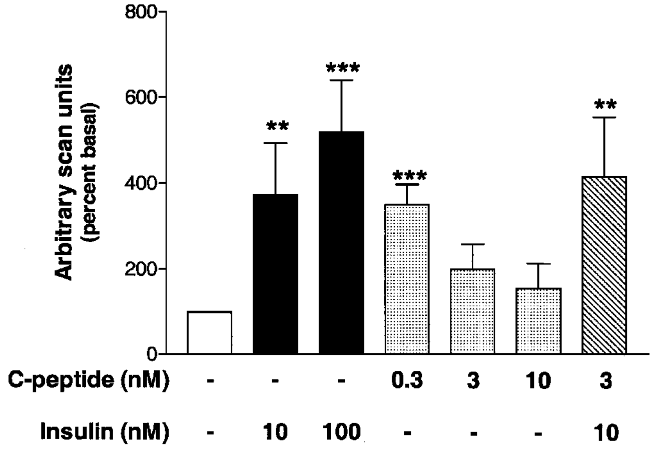

**FIGURE 4**

Activation of MAPK by C-peptide in L6 myoblasts and 3T3-L1 cells. Cells were incubated with insulin, C-peptide, and a combination of insulin and C-peptide for 10 minutes. Cell lysates were separated on Ready gel, transferred to nitrocellulose membrane, and immunoblotted with anti–phospho-MAPK antibody. Quantitation of data (4 separate experiments) is shown as mean ± SEM for each condition normalized for control (=100%). Reproduced from Grunberger et al., 2001, by permission of *Diabetologia*. $^{***}P \ll .001$, $^{**}P < .01$, $^{*}P < .05$ versus control.

C-peptide (0.1 and 3 nM) increased the Rsk activity over that achieved with 10 nM insulin alone.

Biological actions of insulin in skeletal muscle are mediated by a complex interplay of multiple signaling cascades (Virkamäki et al., 1999). One of these insulin-activated signaling pathways involves stimulation of phosphorylation and activation of MAPK or extracellular regulated kinase (ERK). Both insulin and C-peptide (between 0.3 and 10 nM) increased phosphorylation of MAPK in L6 myoblasts and in differentiated 3T3-L1 adipocytes (Figure 4). Incubations with either insulin or C-peptide did not significantly alter ERK protein expression in those cells. Incubation with C-peptide led to a 1.5- to 4-fold increase in L6 myoblasts and 3T3-L1 adipocytes when the results were expressed as phosphorylated MAPK/ERK2 ratio.

To assess the potential role of PI3Ks in C-peptide stimulation of glycogen synthesis in L6 myoblasts, glycogen synthesis was assessed in the presence of a PI3K inhibitor. Wortmannin (at 100 nM) completely abolished the C-peptide–, insulin- as well as the combination of C-peptide/insulin–stimulated glycogen synthesis (Figure 5). These data indicate involvement of wortmannin-sensitive class 1A or 3 PI3Ks (Fruman et al., 1998) in the C-peptide–stimulated glycogen synthesis. In an insulin-responsive, differentiated 3T3-L1 adipocyte system used as a control system, insulin stimulated the PI3K activity associated with tyrosine-phosphorylated proteins. C-peptide (100 nM) increased (~2-fold) PI3K activity in these anti-phosphotyrosine immunoprecipitates. C-peptide at concentrations of 1, 10, and 100 nM increased PI3K activity in a dose-dependent manner. When the assays were done in the presence of wortmannin, PI3K activity was completely abolished. The combination of submaximal concentrations of C-peptide (0.5 nM) with submaximal insulin (1 and 10 nM) increased PI3K activity above that seen with insulin (10 nM) alone. C-peptide increased the activation of PI3K by about 60%. It has to be noted, though, that this assay only assesses the activity of that small portion of PI3K that associates with anti-phosphotyrosine immunoprecipitates (about 1% to 4% of the total PI3K activity). Unlike wortmannin, which inhibited both insulin and C-peptide glycogen synthesis, pertussis toxin had no such effect in L6 myoblasts (Figure 5).

To study the upstream mediator(s) of C-peptide–induced PI3K activation. L6 myoblasts were treated with C-peptide (0.3 and 3 nM) or insulin (10 and 100 nM), corresponding to the submaximal and maximal effective doses, respectively, as determined in the glycogen synthesis and amino acid uptake experiments (Figures 1 and 2). Cell lysates were immunoprecipitated with anti–insulin receptor substrate-1 (IRS-1) antibody and the

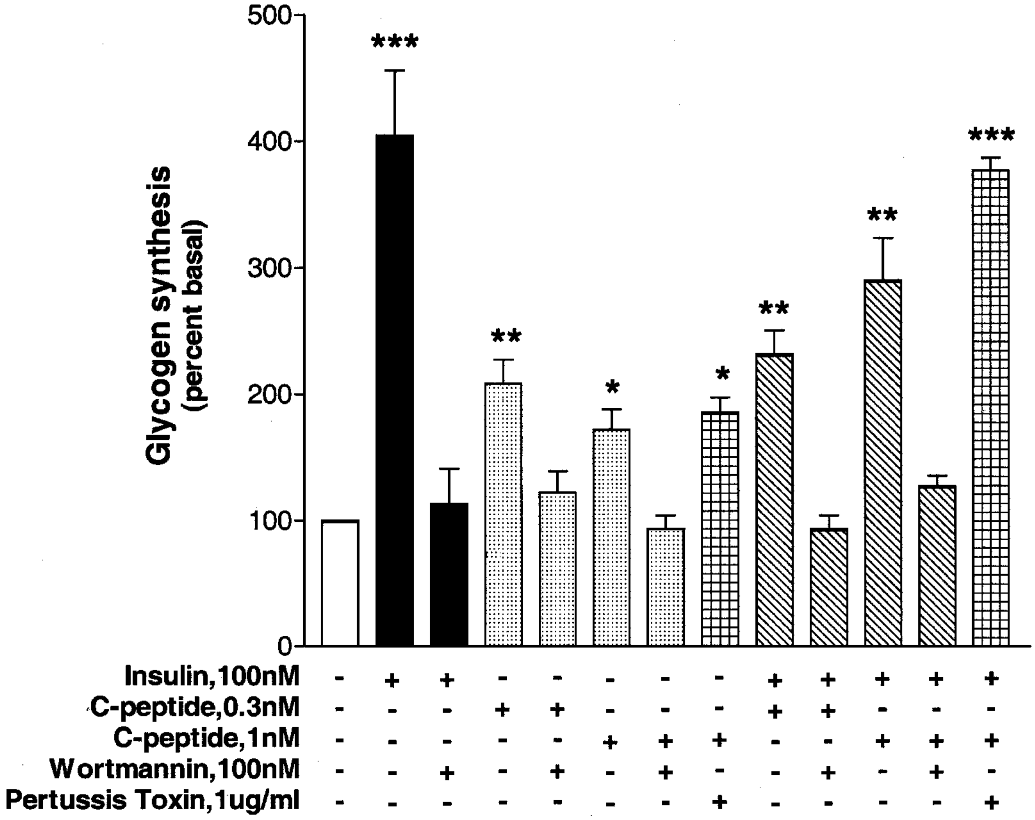

**FIGURE 5**

C-peptide–stimulated glycogen synthesis in L6 myoblasts is wortmannin sensitive but pertussis toxin independent. Monolayers of confluent L6 myoblasts were serum-starved for 16 hours, pretreated with either wortmannin (100 nmol/L) for 30 minutes or with pertussis toxin (1 $\mu$g/mL) for 4 hours and then incubated with insulin (100 nmol/L), with C-peptide (0.3 nmol/L), or with insulin and C-peptide combinations for 1 hour. Glycogen synthesis was assessed by measuring D-[$^{14}$C]glucose incorporation into glycogen. Results are plotted as mean ± SEM of 4 separate experiments (each done in quadruplicate) and normalized with respect to control (=100%). Reproduced from Grunberger et al., 2001, by permission of *Diabetologia*. $^{***}P < .001$, $^{**}P < .01$, $^{*}P < .05$ versus control.

resulting immunocomplexes were subjected to immunoblotting with anti-phosphotyrosine antibody. C-peptide at both 0.3 and 3 nM also significantly increased tyrosine phosphorylation of IRS-1. These data suggest that activation of IRS-1 is involved in C-peptide signaling pathway.

Binding to its receptor and activation of the insulin receptor tyrosine kinase activity (IR-TKA) initiate the bioeffects of insulin. Because C-peptide increased glycogen synthesis and amino acid uptake in insulin-responsive cells, the ability of C-peptide to stimulate TKA in solubilized L6 receptors was tested. C-peptide by itself increased TKA of the extracts in a dose-dependent manner. The bell-shaped concentration response, with maximum effects occurring between 1 and 10 nM, is depicted in Figure 6*A*. The statistically significant increase in TKA caused by C-peptide was comparable to that stimulated by 10 nM insulin. Concentrations of C-peptide leading to the maximum TKA were thus in the range seen physiologically and in the glycogen synthesis and amino acid uptake experiments (Figures 1 and 2). Comparing with submaximally effective insulin (10 nM) concentration, the combination of insulin and C-peptide increased TKA of the L6 myoblast membrane extracts over that of either ligand alone (Figure 6*B*). These results suggest that the insulinomimetic effects of C-peptide in L6 cells could be initiated by activation of TKA. To determine whether IR contributes the TKA stimulated in these experiments by C-peptide, immunoprecipitated IRs (from the wheat germ agglutinin (WGA)-purified membrane preparation using an antibody against IR-$\beta$-subunit) were used in an in vitro kinase assay. C-peptide (0.3 and 3 nM) exerted a 2-fold stimulation of IR TKA. Combination of submaximal insulin (10 nM) and C-peptide (0.3 nM) concentrations increased IR TKA to the same degree as either ligand alone. C-peptide (0.3 and 3 nM) increased IR phosphorylation on tyrosine residues 3- to 4-fold in L6 myoblasts. These data suggest that the

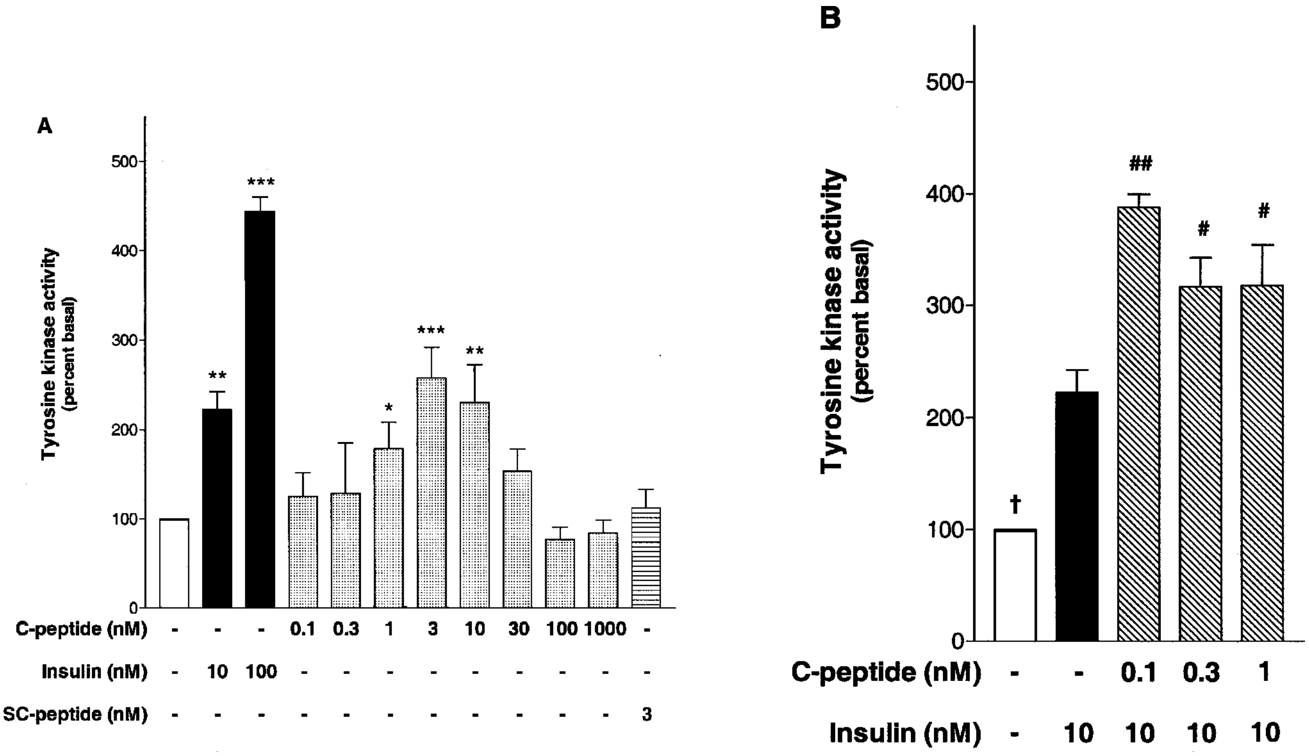

FIGURE 6

C-peptide activates tyrosine kinase in L6 myoblasts. (*A*) Receptors partially purified from L6 plasma membranes were incubated with insulin, C-peptide, or scrambled C-peptide. (*B*) Receptors were incubated with combinations of insulin and C-peptide. Tyrosine kinase activity of the receptors was assayed by phosphorylation of the synthetic substrate poly ($Glu^4Tyr^1$) in the presence of [$\gamma$-$^{32}$P]ATP. Results are shown as mean ± SEM of 3 separate experiments (each done in quadruplicate) and normalized with respect to control (=100%). Reproduced from Grunberger et al., 2001, by permission of *Diabetologia*. $^{***}P < .001$, $^{**}P < .01$, $^{*}P < .05$ versus control; $^{\dagger}P < .05$ versus every sample, $^{\#}P < .05$, $^{\#\#}P < .01$ versus insulin (10 nmol/L).

C-peptide–induced TKA could be at least partly contributed by IR present in the L6 membrane preparations.

Thus, C-peptide, at physiological concentrations, mimics such insulin effects as glycogen synthesis and amino acid uptake in rat muscle cells. C-peptide also stimulates 3-*O*-methylglucose transport in human skeletal muscle strips (Zierath et al., 1991, 1996). C-peptide, at physiological concentrations (generally between 0.3 and 3 nM), mimics qualitatively the effects of insulin. An exception occurred in the case of Akt (PKB), where C-peptide had no effect. Akt is believed to be necessary for the insulin-induced activation of glycogen synthesis in L6 myotubes (Takata et al., 1999). Stimulation of glycogen synthase by insulin is mediated by dephosphorylation through activation of glycogen-associated protein phosphatase (PP1G) and inhibition of GSK3, which, in turn, is inhibited by phosphorylation. There are at least 3 plausible kinases that could phosphorylate GSK3 or PP1G: p70S6 kinase, p90Rsk, and Akt. C-peptide stimulates glycogen synthesis without activating Akt. Whether this response is mediated by a p70S6k- and/or p90Rsk-dependent (Shepherd et al., 1995) and Akt-independent pathway(s) is being investigated. Because of the presence of additional mechanisms (e.g., stimulation of PPG1) for regulation of glycogen synthesis, only qualitative comparisons should be drawn at this time between our data on C-peptide's effect on glycogen synthesis and GSK3 phosphorylation.

Given the reported cellular and tissue effects of C-peptide and its beneficial effects in type 1 diabetic patients and in animal models (Wu et al., 1996; Sjokvist et al., 1998; Sima et al., 1999; Sima, 2003a, 2003b; Johansson et al., 1992; Ekberg et al., 2003), the question still remains as to the molecular underpinnings for these observations. There are at least 4 distinct possibilities for explaining our data (Figure 7). First, C-peptide effects could result from direct binding to and activation of a specific C-peptide receptor. A putative receptor, demonstrated by Rigler et al. (1999), is proposed to be a surface entity coupled to its signal transduction pathway(s) via a G protein. The latter hypothesis is based on the demonstration of blunting the C-peptide effects by pertussis toxin. The data in L6 myoblasts do not

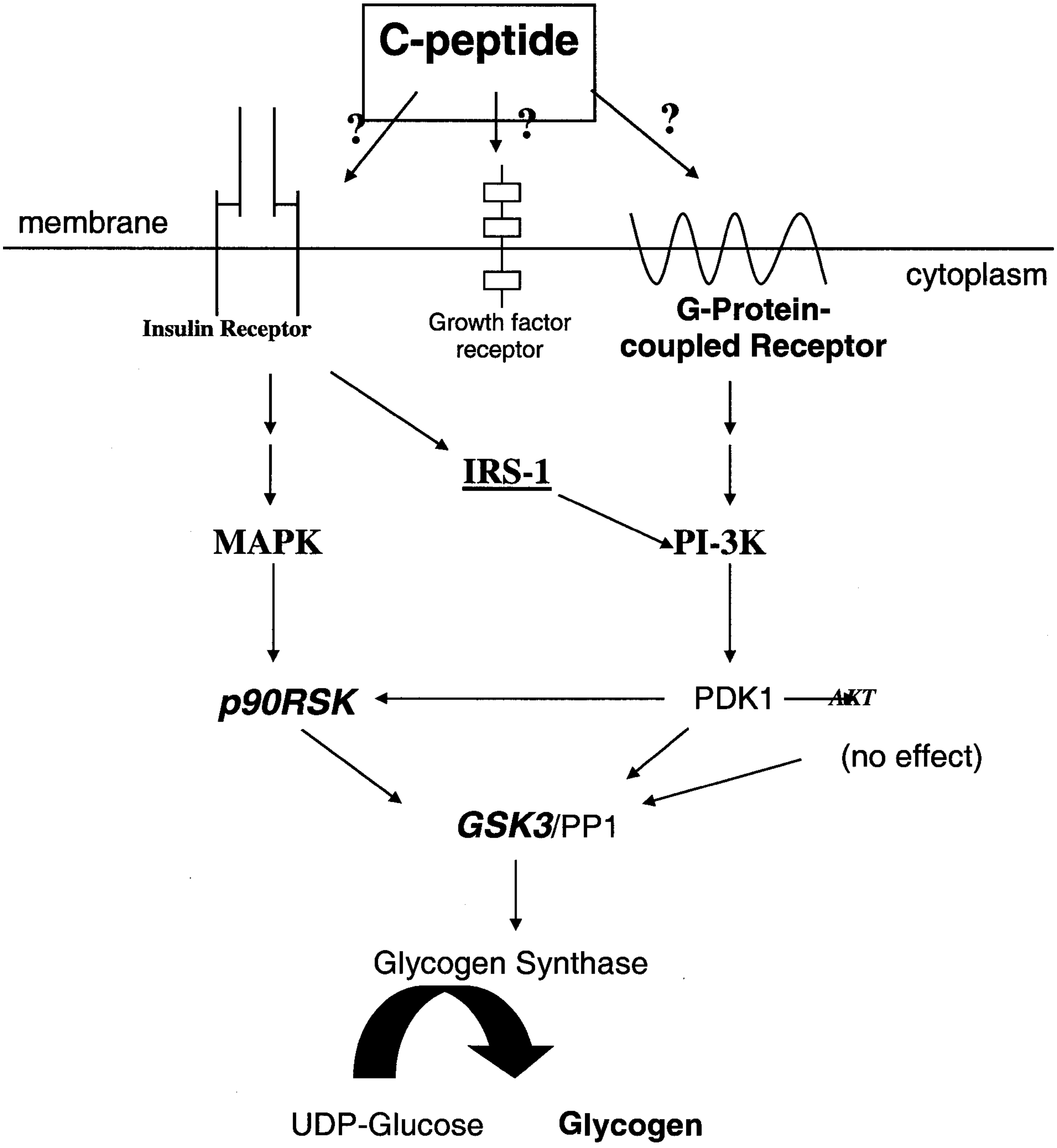

FIGURE 7
Possible mechanisms of C-peptide effects.

indicate any effect of pertussis toxin on C-peptide–stimulated glycogen synthesis, however. The second possibility is that C-peptide activates the IR. The lack of C-peptide stimulation of glycogen synthesis in the parental rat 1 fibroblasts (which contain few IR) but robust effects in HIRcB cells (overexpressing human IR) support a role of IR in mediating the C-peptide effect. Given the structural differences between insulin and C-peptide, the interaction with IR would need to occur with an $\alpha$-subunit domain distinct from that responsible for insulin binding to IR. Alternatively, a plasma membrane perturbation caused by C-peptide could lead indirectly to a conformational change of the IR, leading to activation of its $\beta$-subunit, i.e., its autophosphorylation and activation of tyrosine kinase. The fact that only submaximal concentrations of both C-peptide and insulin are additive suggests sharing of at least some elements of the signaling pathways used by these ligands. Interestingly,

Jensen and Messina (1999) showed, in smooth muscle arterioles, that the effect of C-peptide on arteriolar dilation was potentiated in the presence of low insulin concentrations alone. They concluded that the C-peptide interaction with insulin is biologically important and concentration dependent. This interaction might be missing in patients treated solely with insulin replacement. The third possibility is a combination of the two above: interaction of C-peptide with its cognate membrane receptor and with a specific domain of the IR. A fourth option is an interaction of C-peptide with another cell surface receptor such as the insulin-like growth factor (IGF)-1 receptor. IGF-1 and C-peptide share structural domains, which could form the basis for cross-talk between their signaling pathways. We have already shown that C-peptide replacement normalizes both IGF and IGF-1 receptor expression (Li et al., 2000; Sima et al., 2001).

The respective insulin and C-peptide concentration-response curves deserve mention. Typically, 10 nM insulin is necessary to document statistically significant responses in in vitro systems. Higher insulin concentrations increase the effect, with 100 to 1000 nM resulting in the maximal stimulation of a response tested in the various assays. In contrast, considerably less C-peptide, on a molar basis, is sufficient to elicit insulin-like responses, even though in absolute terms, these are less robust than those seen with insulin alone. Peaks of C-peptide responses are reached at ~0.3 to 3 nM. Interestingly, half saturation of the putative C-peptide receptor occurs at 0.3 nM and full saturation at 0.9 nM of the peptide (Rigler et al., 1999), i.e., concentrations found fully effective in the L6 cell system. In contrast to insulin, higher doses of C-peptide (>10 nM) result in blunting of the stimulatory responses. Any extrapolation of these observations into mammalian physiology is, of course, premature. One wonders, however, whether the additivity of low C-peptide and low insulin concentrations could be advantageous for fuel storage. Conversely, in the postprandial situation, in the presence of exuberant insulin release, the concomitant higher C-peptide levels would serve to blunt insulin's peripheral effects.

### C-PEPTIDE EFFECT ON PROTEIN TYROSINE PHOSPHATASE IN L6 RAT SKELETAL MYOBLASTS

Based on the finding that C-peptide mimicked at least partly the molecular details of insulin signal transduction, Li et al. (2001) tested the hypothesis that C-peptide affected the activity of protein tyrosine phosphatase (PTP). C-peptide indeed inhibited PTP activity in rat skeletal muscle cells in a dose-dependent manner. Maximum inhibition appeared at 3 nM of C-peptide, the same concentration as the maximum stimulatory effect of C-peptide on glycogen synthesis. Scrambled C-peptide sequence had no effect on PTP activity. Autophosphorylation of the IR and activation of IRS-1 were enhanced by C-peptide. These findings thus provide evidence for possible mechanisms underlying the insulinomimetic effects of C-peptide. C-peptide could be envisioned to interact with the IR or could cross-talk with the IR signaling pathway at a proximal point. Of course, another possibility would include C-peptide first interacting with its specific G protein–coupled receptor, leading to a consequent cross-talk with the IR signal transduction pathway.

### EFFECTS OF C-PEPTIDE ON NEURONAL APOPTOSIS

In another attempt to establish the molecular mechanisms responsible for the salutary C-peptide effects on diabetic neuropathy, the group of Sima studied human neuroblastoma SH-SY5Y cells (Li et al., 2003; Sima, 2003a). They demonstrated that C-peptide stimulated cell proliferation and neurite outgrowth. Cell numbers increased with duration of treatment, reaching maximum at 4 days. Cell number increase was dose dependent, with maximum effect observed at 3 nM of C-peptide. Scrambled C-peptide did not affect cell proliferation. As expected, insulin, at 4 nM, was also a stimulator of cell proliferation. Significantly, insulin's effect was enhanced by the addition of physiological doses (1 to 3 nM) of C-peptide. IGF-1 (at 1 nM) also increased cell numbers and its effect was enhanced by 1 to 10 nM of C-peptide. For neurite outgrowth, results were mostly analogous to the cell proliferation part of the experiments. The addition of C-peptide (3 to 10 nM) increased the neurite outgrowth over and above that seen with either insulin or C-peptide alone. Interestingly, addition of C-peptide did not enhance the effect of IGF-1 (1 nM) alone. Scrambled-sequence C-peptide again had no effect on the neurite outgrowth. These cellular effects of C-peptide were likely the result of increased antiapoptosis. The authors examined glucose-induced apoptosis (at 5 to 200 mM range). The percentage of apoptotic cells grew with increased glucose concentrations. Effects of C-peptide on glucose-induced apoptosis were tested at 100 mM glucose concentration. C-peptide by itself (at 3 nM), in contrast to insulin (at 4 nM), had no protective effect on glucose- or mannitol-induced apoptosis. The combination of insulin and C-peptide led to enhancement of the antiapoptotic effects of either insulin or C-peptide alone. Results were the same when apoptosis was examined by nuclear condensation. Combination of C-peptide and insulin resulted in a more dramatic protection against apoptosis than the effect of insulin alone. Neither insulin nor C-peptide affected expression of $Bcl_2$ or nuclear factor (NF)-$\kappa$B. The combination of C-peptide and insulin, however, significantly increased the expression of both $Bcl_2$ and NF-$\kappa$B. Likewise, the combination of C-peptide and insulin enhanced translocation of NF-$\kappa$B to the nuclei over the effect of insulin alone.

Interestingly, C-peptide, in the concentration of 3 nM and in presence of 4 nM insulin, significantly enhanced autophosphorylation of the IR. Further, the combination of C-peptide and insulin in this cell system increased phosphorylation of p38 MAPK, stimulated PI3K activity in antiphosphotyrosine immunoprecipitates, increased the expression and translocation of NF-$\kappa$B, enhanced expression of $Bcl_2$, and reduced phosphorylation of JNK. All of these effects were significantly higher than those seen in presence of insulin alone. C-peptide by itself had no effects on any of these signaling elements. Finally, no enhancement of tyrosine phosphorylation of a related receptor, that for IGF-1, was detected in presence of the combination of C-peptide and insulin. The authors concluded that the antiapoptotic effect of C-peptide enhances that of insulin, but through a different mechanism from that induced by IGF-1. Given their data, C-peptide could possibly lead to antiapoptotic action by increased phosphorylation of several cellular proteins, including those involved in the insulin signaling pathways.

Therefore, these effects of C-peptide in human neuroblastoma cells, occurring in the presence of submaximal concentrations of insulin, are similar to those reported by Jensen and Messina (1999) showing that the concentration-dependent arteriolar dilatation by C-peptide occurs only in the presence of insulin.

In summary, it is clear that C-peptide alone or in combination with low insulin concentrations mimics several of insulin's biological effects. In insulin-sensitive L6 myoblasts, differentiated myocytes, adipocytes, and neuroblastoma cells, the signal transduction pathways activated by C-peptide are similar to those used by insulin. The receptor through which the C-peptide activities are transmitted, however, remains elusive. The elucidation of its nature and cloning and details of the interaction between the insulin and C-peptide signaling pathways pose important challenges for the immediate future.

### REFERENCES

Ekberg, K., Brismar, T., Johansson, B.-L., Jonsson, B., Lindström, P., and Wahren, J. (2003) Amelioration of sensory nerve dysfunction by C-peptide in patients with type 1 diabetes. *Diabetes,* **52,** 536–542.

Fruman, D. A., Meyers, R. E., and Cantley, L. C. (1998) Phosphoinositide kinases. *Annu. Rev. Biochem.,* **67,** 481–507.

Grunberger, G., Qiang, X., Mathews S. T., Sbrissa, D., Shisheva, A., and Sima, A. A. F. (2001) Molecular basis for the insulinomimetic effects of C-peptide. *Diabetologia,* **44,** 1247–1257.

Halse, R., Rochford, J. J., McCormack, J. G., Vandenheede, J. R., Hemmings, B. A., and Yeaman, S. J. (1999) Control of glycogen synthesis in cultured human muscle cells. *J. Biol. Chem.,* **274,** 776–780.

Holman, G. D., and Kasuga, M. (1997) From receptor to transporter: Insulin signaling to glucose transport. *Diabetologia,* **40,** 991–1003.

Ido, Y., Vindigni, A., Chang, K., Stramm, L., Chance, R., Heath, W. F., DiMarchi, R. O., DiCera, E., and Williamson, J. R. (1997) Prevention of vascular and neural dysfunction in diabetic rats by C-peptide. *Science,* **277,** 563–566.

Jensen, M. E., and Messina, E. J. (1999) C-peptide induces a concentration-dependent dilation of skeletal muscle arterioles only in presence of insulin. *Am. J. Physiol* **276** *(Heart Circ. Physiol.* **45**), H1223–H1228.

Johansson, B. L., Sjöberg, S., and Wahren, J. (1992) The influence of human C-peptide on renal function and glucose utilization in type-1 diabetic patients. *Diabetologia,* **35,** 121–128.

Kitamura, T., Kimura, K., Jung, B. D., Makondo, K., Okamoto, S., Canas, X., Sakane, N., Yoshida, T., and Saito, M. (2001) Proinsulin C-peptide rapidly stimulates mitogen-activated protein kinases in Swiss 3T3 fibroblasts: Requirement of protein kinase C, phosphoinositide 3-kinase and pertussis toxin-sensitive G protein. *Biochem. J.,* **355,** 123–129.

Kitamura, T., Kimura K., Jung B. D., Makondo, K., Sakane, N., Yoshida, T., and Saito, M. (2002) Proinsulin C-peptide activates cAMP response element-binding proteins through the p38 mitogen-activated protein kinase pathway in mouse lung capillary endothelial cells. *Biochem. J.,* **366,** 377–744.

Li, Z.-G., Qiang, X., Sima, A. A. F., and Grunberger, G. (2001) C-peptide attenuates protein tyrosine phosphatase activity and enhances glycogen synthesis in L6 myoblasts. *Biochem. Biophys. Res. Commun.,* **280,** 615–619.

Li, Z., Zhang, W., Grunberger, G., and Sima, A. A. F. (2000) C-peptide corrects IGF's and prevents apoptosis in type 1 BB/Wor rat hippocampus. *Diabetes,* **49(Suppl. 1),** A229.

Li, Z.-G., Zhang, W., and Sima, A. A. F. (2003) C-peptide enhances insulin-mediated cell growth and protection against high glucose-induced apoptosis in SH-SY5Y cells. *Diabetes. Metab. Res. Rev.,* **19,** 375–385.

Ohtomo, Y., Aperia, A., Sahlgren, B., Johansson, B.-L., and Wahren, J. (1996) C-peptide stimulates rat renal tubular $Na^+$, $K^+$ ATPase activity in synergism with neuropeptide Y. *Diabetologia,* **39,** 199–205.

Rigler, R., Pramanik, A., Jonasson, P., Kratz, G., Jansson, O., Nygren, P.-Å., Ståhl, S., Ekberg, K., Johansson, B.-L., Uhlén, S., Uhlén, M., Jörnvall, H., and Wahren, J. (1999) Specific binding of proinsulin C-peptide to human cell membranes. *Proc. Natl. Acad. Sci. U. S. A.,* **96,** 13318–13323.

Shepherd, P. R., Nave, B. T., and Siddle, K. (1995) Insulin stimulation of glycogen synthesis and glycogen synthase activity is blocked by wortmannin and rapamycin in 3T3-L1 adipocytes: Evidence for the involvement of phosphoinositide 3-kinase and p70 ribosomal protein-S6 kinase. *Biochem. J.,* **305,** 25–28.

Sima, A. A. F. (2003a) New insights into the metabolic and molecular basis for diabetic neuropathy. *Cell. Mol. Life Sci.,* **60,** 2445–2464.

Sima, A. A. F. (2003b) C-peptide and diabetic neuropathy. *Expert Opin. Invest. Drugs,* **12,** 1471–1488.

Sima, A. A. F., Srinivas, P. R., Kommaraju, S., Verma, S., and Grunberger, G. (1998) Enhancement of insulin receptor activity by C-peptide. *Diabetologia,* **41(Suppl. 1),** A177.

Sima A. A. F., Zhang, W., Sugimoto, K. Henry, D., Li, Z.-G., Wahren, J., and Grunberger, G. (2001) C-peptide prevents and improves chronic type 1 diabetic polyneuropathy in the BB/Wor rat. *Diabetologia,* **44,** 889–897.

Sima, A. A. F., Zhang, W., Xu, G., Wahren, J., and Sugimoto, K. (1999) Long-term effect of C-peptide treatment on type-1 diabetic neuropathy. *Diabetes,* **48(Suppl. 1),** A149.

Sjokvist, M., Huang, W., and Johansson, B. (1998) Effects of C-peptide on renal function at early stage of experimental diabetes. *Kidney Int.,* **54,** 758–764.

Steiner, D. F. (1967) Evidence for a precursor in the biosynthesis of insulin. *Trans. N. Y. Acad. Sci.,* **30,** 60–68.

Takata, M., Ogawa, W., Kitamura, T., Hino, Y., Kuroda, S., Kotani, K., Klip, A., Gingras, A., Sonenberg, N., and Kasuga, M. (1999) Requirement for Akt (protein kinase B) in insulin-induced activation of glycogen synthase and phosphorylation of 4E-BP1 (PHAS-1). *J. Biol. Chem.,* **274,** 20611–20618.

Tsimaratos, M., Roger, F., Chabardès, D., Mordasini, D., Hasler, V., Doncet, A., Martin, P. Y., and Feraille, E. (2003) C-peptide stimulates $Na^+$, $K^+$ ATPase activity via PKC alpha in rat medullary thick ascending limb. *Diabetologia,* **46,** 124–131.

Virkamäki, A., Ueki, K., and Kahn, C. R. (1999) Protein-protein interaction in insulin signaling and the molecular mechanisms of insulin resistance. *J. Clin. Invest.,* **103,** 931–943.

Wahren, J., Ekberg, K., Johansson, J., Henriksson, M., Pramavik, A., Johansson, B.-L., Rigler, R., and Jörnvall, H. (2000) Role of C-peptide in human physiology. *Am. J. Physiol.,* **278** *(Endocrinol. Metab.*), E759-E768.

Wu, W., Oshida, Y., Yang, W. P., Li, L., Ohsawa, I., Sato, J., Iwao, S., Johansson, B.-L., Wahren, J., and Sato, Y. (1996) Effect of C-peptide administration on whole body glucose utilization in STZ-induced diabetic rats. *Acta Physiol. Scand.,* **157,** 253–258.

Zierath, J. R., Galuska, D., and Johansson, B.-L., and Wallberg-Henriksson, H. (1991) Effect of human C-peptide on glucose transport in *in vitro* incubated human skeletal muscle. *Diabetologia,* **34,** 899–901.

Zierath, J. R., Handberg, A., Tally, M., and Wallberg-Henriksson, H. (1996) C-peptide stimulates glucose transport in isolated human skeletal muscle independent of insulin receptor and tyrosine kinase activation. *Diabetologia,* **39,** 306–313.

